# Psoas Muscle Sarcopenia Predicts Brain Volume Loss on MRI in 7,149 Individuals

**DOI:** 10.1002/alz.093444

**Published:** 2025-01-09

**Authors:** Cyrus A. Raji, Somayeh Meysami, Sam Hashemi, Saurabh Garg, Nasrin Akbari, Ahmed Gouda, Yosef Gavriel Chodakiewitz, Thanh Duc Nguyen, Kellyann Niotis, David A. Merrill, Rajpaul Attariwala

**Affiliations:** ^1^ Mallinckrodt Institute of Radiology, Washington University in St. Louis, St. Louis, MO USA; ^2^ Pacific Brain Health Center, Pacific Neuroscience Institute and Foundation, Santa Monica, CA USA; ^3^ Saint John's Cancer Institute at Providence Saint John's Health Center, Santa Monica, CA USA; ^4^ Prenuvo, Vancouver, BC Canada; ^5^ Voxelwise Imaging Technology, Vancouver, BC Canada; ^6^ Vigilance Health Imaging Network Inc., Vancouver, BC Canada; ^7^ Early Medical, Austin, TX USA; ^8^ The Institute for Neurodegenerative Diseases (IND) Florida, Boca Raton, FL USA; ^9^ David Geffen School of Medicine at UCLA, Los Angeles, CA USA; ^10^ AIM Medical Imaging, Vancouver, BC Canada

## Abstract

**Background:**

Sarcopenia has been linked to brain atrophy and there is lack of information on specific muscle groups that may contribute to this link. The psoas muscles are sensitive to sarcopenia and thus may sensitively relate to brain aging and Alzheimer disease risk.

**Method:**

This study utilized 7,149 healthy individuals across four sites (Mean age 53.06 ± 12.95 years, age range 18‐97 years; 48% women; 52% men; 38% non‐white) were scanned on 1.5T MR machines with a whole‐body protocol. Whole body sequences included coronal T1 for segmentation of psoas muscle volumes. Deep learning with FastSurfer on 3D T1 volumetric MPRAGE trained on 134 participants aged 27‐66 segmented 96 brain regions. Partial correlation analysis was done on psoas muscle volumes and brain regions controlling for sex and total intracranial volume to determine if lower psoas muscle volumes correlated to brain atrophy. Multiple comparisons were accounted for using the Bonferroni Method.

**Result:**

Lower psoas muscle volumes demonstrated a statistically partial correlation to lower gray matter (rp = ‐0.164, p = 2.66×10^−43^) and white matter volumes (rp = ‐0.171, p = 4.39×10^−47^). The frontal lobe exhibited a negative correlation (rp = ‐0.172, p = 1.30×10^−47^), identical to the temporal lobe (rp = ‐0.172, p = 1.21×10^−47^). Negative correlations were also noted in the parietal lobe (rp = ‐0.119, p = 8.72×10^−23^), and occipital lobe (rp = ‐0.139; p = 2.89×10^−31^). The hippocampus also showed a statistically significant negative correlation (rp = ‐0.145, p = 7.65×10^−34^), as did the posterior cingulate (rp = ‐0.162, p = 5.20×10^−42^) and the precuneus (rp = ‐0.101, p = 1.34×10^−16^). The cerebral ventricles showed a non‐significant negative correlation (rp = ‐0.025, p = 0.345).

**Conclusion:**

We demonstrate a significant correlation between lower psoas muscle volume and brain volume loss, including AD risk regions such as the hippocampus and precuneus. These MRI findings underscore the importance of muscle health in relation to brain integrity. The volume of the psoas, which is involved in basic leg flexion activities such as walking, could be a crucial indicator of brain health and Alzheimer risk.

